# Cytotoxic Aporphine Alkaloids from Leaves and Twigs of *Pseuduvaria trimera* (Craib)

**DOI:** 10.3390/molecules19078762

**Published:** 2014-06-25

**Authors:** Wuttikrai Sesang, Sittiporn Punyanitya, Siripit Pitchuanchom, Phansuang Udomputtimekakul, Narong Nuntasaen, Ratana Banjerdpongchai, Benjawan Wudtiwai, Wilart Pompimon

**Affiliations:** 1 Laboratory of Natural Products, Center for Innovation in Chemistry, Faculty of Science, Lampang Rajabhat University, Lampang 52100, Thailand; E-Mails: wuttikrai42222@hotmail.com (W.S.); meen2@hotmail.com (S.P.); Phansuang@yahoo.com (P.U.); 2 Science and Technology Research Institute, Chiang Mai University, Chiang Mai 53000, Thailand; E-Mail: punyanitya.s@gmail.com; 3 The Forest Herbarium, Department of National Park, Wildlife and Plant Conservation, Ministry of Natural Resources and Environment, Bangkok 10220, Thailand; E-Mail: narong_1960@hotmail.com; 4 Department of Biochemistry, Faculty of Medicine, Chiang Mai University, Chiang Mai 53000, Thailand; E-Mails: ratana@chiangmai.ac.th (R.B.); boaunn@gmail.com (B.W.)

**Keywords:** aporphine alkaloid, cytotoxicity, anti-cancer, *Pseuduvaria trimera*, Annonaceae

## Abstract

From ethyl acetate-methanol extracts of leaves and twigs of *Pseuduvaria trimera* a new aporphine alkaloid; 8-hydroxy-1,4,5-trimethoxy-7-oxoaporphine or 8-hydroxyartabonatine C (**1**) was isolated, together with the known 1,2,3-trimethoxy-4,5-dioxo-6a,7-dehydroaporphine (ouregidione, **2**). Their structures were elucidated by a combination of spectral methods; mainly 2D NMR; IR and MS. Compounds **1** and **2** exhibited cytotoxic activity with IC_50_ values of 26.36 ± 5.18 μM and 12.88 ± 2.49 μM, respectively, for human hepatocellular carcinoma HepG2 cells, and 64.75 ± 4.45 and 67.06 ± 3.5 μM, respectively, for human breast cancer MDA-MB231 cells. Both compounds displayed anti-cancer activity but less than that of doxorubicin; a conventional chemotherapeutic drug, the IC_50_ levels of which were 2.21 ± 1.72 and 1.83 ± 0.09 μM for HepG2 and MDA-MB231 cells, respectively.

## 1. Introduction

*P. trimera* belongs to the family Annonaceae, which includes about eight species in Thailand (*P. gardneri*, *P. macrophylla*, *P. monticola*, *P. muitiovulata*, *P. reticulata*, *P. rugosa*, *P. setosa*, *P. trimera*) [1]. It forms evergreen and deciduous broad forests on base lime-stone mountains. *P. trimera* is classified as a tree, 20 m tall, branches pale gray, densely puberulent when young, glabrescent [2]. 

Previous work on plants of this genus have revealed the presence of alkaloids in every *Pseuduvaria* species investigated such as aporphine, 1,2,3-trimethoxy-4,5-dioxo-6a,7-dehydroaporphine and *O*-methylmoschatoline [3], *N*-methylouregidione, liriodenine, oxostephanine [4], pseuduvarines A and B [5], oxoanolobine [6] and 1,2,3-trimethoxy-5-oxonoraporphine (ouregidione) [4,7]. However, no phytochemical investigation of this plant species has been reported to date. *Pseuduvaria* species are traditionally used to treat fever, nausea, headache and stomach ailment and have been mostly studied for alkaloids [8]. In our search for biologically active constituents from this plant, we have subjected its extracts to *in vitro* screening for anticancer activity against two cancer cell lines (human hepatocellular carcinoma HepG2 cells and human breast cancer MDA-MB231cells). The goal of this study was to describe the isolation, structural elucidation and cytotoxicity of aporphine alkaloids from *P. trimera*. 

## 2. Results and Discussion

**Figure 1:**
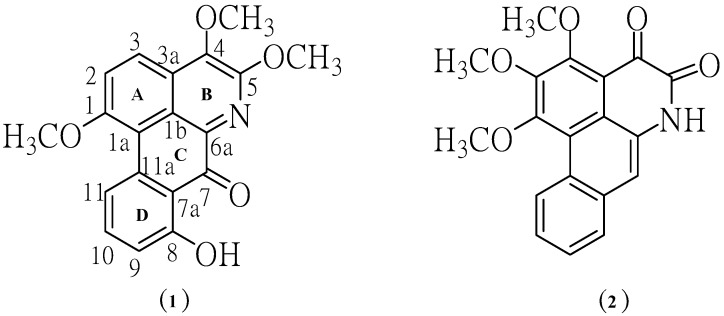
Structures of the isolated aporphine alkaloids.

Phytochemical investigation of the ethyl acetate-methanol extract obtained from the mixture of leaves and twigs of *P. trimera* led to the isolation of two alkaloids, namely 8-hydroxy-1,4,5-trimethoxy-7-oxoaporphine (**1**) and 1,2,3-trimethoxy-4,5-dioxo-6a,7-dehydroaporphine (**2**). The structures of aporphine alkaloids **1** and **2** (Figure 1) were elucidated by spectroscopic methods, including ^1^H-NMR, ^13^C-NMR, UV, IR, MS.

**Figure 2:**
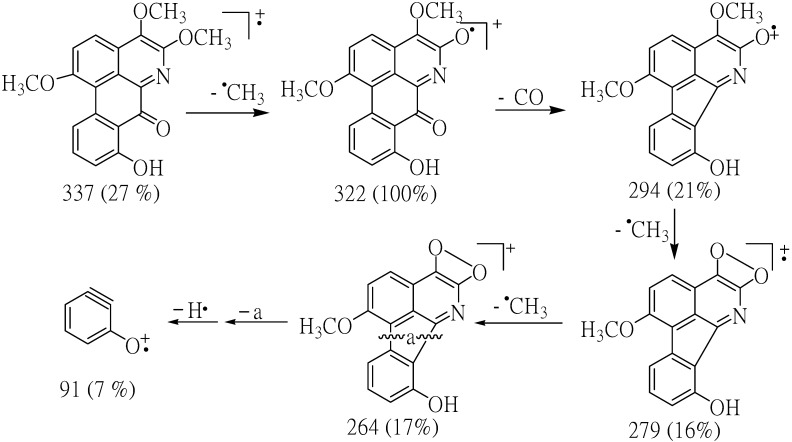
Most important observed fragmentations for compound **1**.

Compound **1** crystallized as orange crystals, m.p. 251–252 °C and its molecular formula was determined to be C_19_H_15_NO_5_ by HR-ESIMS. Moreover, its UV spectrum exhibited absorption peaks at 210, 241, 317 and 417 nm indicating the presence of a highly conjugated system. The IR absorptions for OH (3249 cm^−1^), C=O (1705 cm^−1^), aromatic (1658, 1559, 1508, 1458 cm^−1^) and ether moieties (1281, 1211 cm^−1^) were also observed. From the UV and IR spectral data it was indicated that **1** was an oxoaporphine derivative [9]. The fragment ions at *m/z* 337 (M^+^) and 322 (M^+^-Me) in the mass spectrum of compound **1** indicated the presence of OMe group in the position next to N in the structure. The results from decarbonylation cleavage at *m/z* 294 indicated the presence of carbonyl group in ring C. The ion of *m/z* 91 supported that **1** contained one hydroxyl group in ring D (Figure 2).

**Figure 3:**
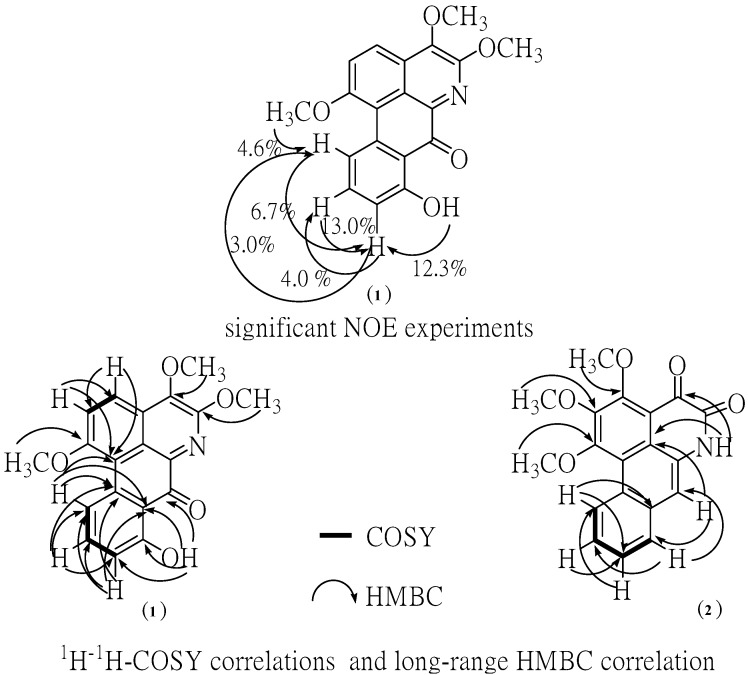
NOE experiment, significant correlations in the COSY and HMBC spectra.

The ^1^H-NMR spectrum of the major alkaloid, in addition to three methoxy (***δ*** 4.10, *s*), (3.99, *s*) and 3.97, *s*) groups, had three obscured signals at ***δ*** 7.57, 7.60 and 7.62, and two multiplet signals at ***δ*** 7.88 and 9.34 in aromatic ring region, so adding together hydroxyl group (12.02, *s*), this accounts for all 15 protons. A downfield singlet proton at ***δ*** 12.02 was assigned to a hydroxyl group at the C-8 position due to the formation of hydrogen bonding with neighboring carbonyl group at C-7. Moreover, the sequential correlations of aromatic proton signals at C-9 (*δ* 7.57), C-10 (*δ* 7.88) and C-11 (*δ* 9.34) on ring D were clearly proven by the COSY spectrum. Additionally, the aromatic proton at C-2 (*δ* 7.60) and C-3 (*δ* 7.62) are also clearly supported by this COSY spectrum and HMBC correlation with C-1a ,C-3 (strong correlation) and C-1a (weak correlation), C-2, respectively (Figure 3). Moreover, the identification of H-9, 10, 11 in ring D was determined by NOE difference spectra. Irradiation of H-8 (OH) signal showed enhancement of the H-9, thus indicating that it definitely has one proton next to the OH group. In addition, irradiation at the methoxy signal of position 1 also showed enhancement of H-11. Accordingly, the H-10 was confirmed at this position since there was NOE enhancement with the H-9 (Figure 3). Correspondingly, the presence of a hydroxyl group in the molecule located in the ring D at C-8 was established on the basis of long-range ^1^H-^13^C correlation of the HO-8 at *δ* 12.02 with the carbon C-9 (*δ* 113.76), C-7 (*δ* 175.55). The ^13^C-NMR spectrum exhibited the presence of three methoxyl group, five methine, and eleven quaternary carbons (Table 1). In comparison with the literature data [10] the keto group at C-7 position usually resonance at *δ* 175 indicating the existence of this carbonyl at the peri-position. 

**Table 1:** ^1^H-NMR (500 MHz), ^13^C-NMR (125 MHz) in DMSO-*d*_6_ **1** and CDCl_3_ for **2**.

Position		8-Hydroxy-1,4,5-trimethoxy-7-oxoaporphine (1)			1,2,3-Trimethoxy-4,5-dioxo-6a,7-dehydroaporphine (2)	
		* *δ* ^1^H m (*J* Hz)		*δ* ^13^C(DEPT)	* *δ* ^1^H m (*J* Hz)	*δ* ^13^C(DEPT)
1		-		159.15 (C)	-	158.66 (C)
1a		-		131.58 (C)	-	116.26 (C)
1b		-		120.21 (C)	-	120.31 (C)
2		7.60 *obsc*.		127.51 (CH)	-	147.54 (C)
3		7.62 *obsc*.		126.87 (CH)	-	160.44 (C)
3a		-		117.65 (C)	-	117.62 (C)
4		-		147.02 (C)	-	175.41 (C)
5		-		157.49 (C)	-	157.63 (C)
6a		-		129.82 (C)	-	128.38 (C)
7		-		175.55 (C)	7.83 *s*	116.0 (CH)
7a		-		119.21 (C)	-	131.77 (C)
8		-		155.80 (C)	7.98 *m*	128.48 (CH)
9		7.57 *obsc*.		113.76 (CH)	7.65 *obsc*.	127.57 (CH)
10		7.88 *m*		128.17 (CH)	7.66 *obsc*.	127.44 (CH)
11		9.34 *m*		126.81 (CH)	9.5 *m*	127.24 (CH)
11a		-		125.91 (C)	-	121.23 (C)
1-OMe		4.10 *s*		61.09 (CH_3_)	4.17 *s*	62.08 (CH_3_)
2-OMe		-		-	4.10 *s*	61.74 (CH_3_)
3-OMe		-		-	4.21 *s*	61.17 (CH_3_)
4-OMe		3.99 *s*		61.59 (CH_3_)	-	-
5-OMe		3.97 *s*		61.65 (CH_3_)	-	-
8-OH		12.02 *s*		-	-	-
N-H		-		-	11.77 *s*	-

* *δ* in ppm from TMS [coupling constants (*J*) in Hz are given in parentheses]; *obsc*. = obscure signal.

In addition, the HMBC spectra gave further support for carbonyl group by correlations with hydroxy proton. The methoxy group at C-1 position of ring A was confirmed by NOE enhancement with H-11. In addition, the methoxy group at C-4 and 5 position of ring B confirmed by direct comparison of those chemical shifts with the ^13^C-NMR of artabonatine C, a compound isolated from *Artabotrys uncinatus* [10]. According to the ^1^H and ^13^C-NMR 1D/2D data this compound was identified as the oxoaporphine alkaloid. On the basis of the above data, the structure for **1** was formulated as 8-hydroxy-1,4,5-trimethoxy-7-oxoaporphine. It is a new alkaloid and does not appear to have been previously isolated from this species. Chromatographic separation of the ethyl acetate extract afforded compound **2**. This compound is isomeric with compound **1**. The structure was established by comparison of their UV, IR, EIMS, and 1D, 2D NMR data with the literature data [4,7,11]. 

**Figure 4:**
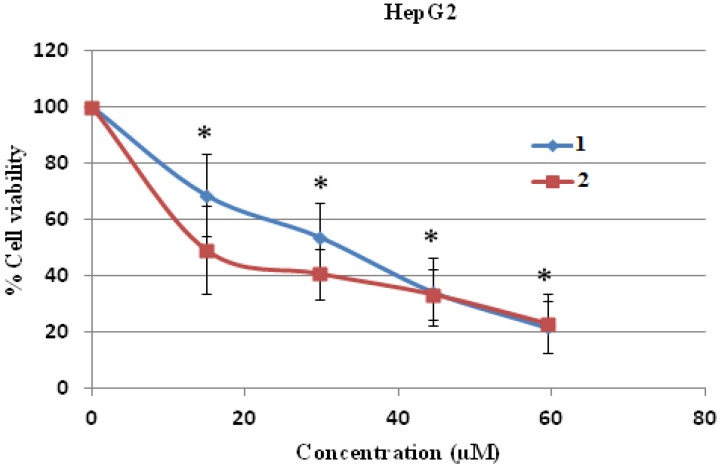
Cell cytotoxicity of **1** and **2** on human hepatocellular carcinoma HepG2 cells. HepG2 cells were treated with **1** and **2** at various concentrations for 24 h and the cell viability was determined by MTT assay. * *p* < 0.05, compared to control of both compound **1** and **2**.

**Figure 5:**
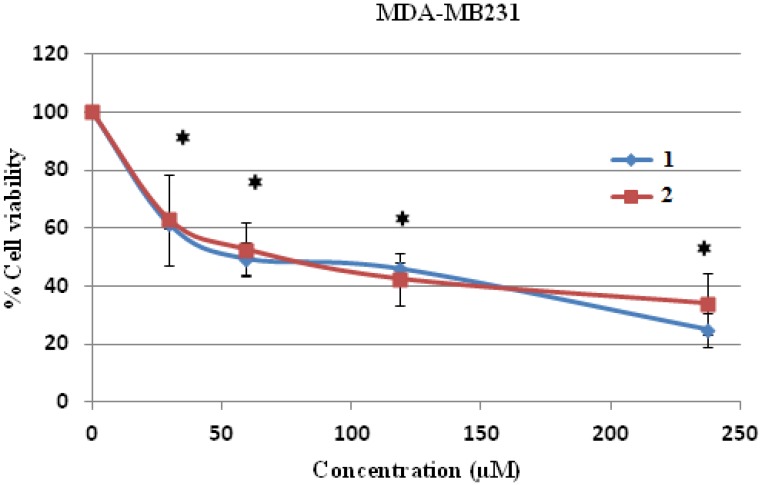
Cell cytotoxicity of **1** and **2** on human breast cancer MDA-MB231cells. MDA-MB231 cells were treated with **1** and **2** at various concentrations for 24 h and the cell viability was determined by MTT assay. * *p* < 0.05, compared to control of both compound **1** and **2**.

The cytotoxicity test was performed by the MTT assay and it was found that compounds **1** and **2** were toxic to both human hepatocellular carcinoma HepG2 and human breast cancer MDA-MB231 cell lines dose dependently, and these toxicities were statistically and significantly different at the IC_10_, IC_20_ and IC_50_ levels when compared to control (without treatment) (Figure 4 and Figure 5). Compound **2** was more cytotoxic to HepG2 cells than compound **1**, with an IC_50_ concentration of 12.88 ± 2.49 μM compared to 26.36 ± 5.18 μM, respectively, and the IC_50_ values of both compounds were significantly different when compared to each other (Table 2). Both IC_50_ levels of compound **1** and **2** were statistically different compared to those of doxorubicin in both cells (Table 2 and Table 3). 

However, when human breast cancer MDA-MB231 cells were treated with both compounds, it was shown that both compound **1** and **2** were cytotoxic to the MDA-MB231 cells up to the tested concentration of 240 μM (Figure 5). The IC_50_ levels of both compound **1** and **2** on human breast cancer MDA-MB231 cells were 64.75 ± 4.45 and 67.06 ± 3.5 μM, respectively (Table 3). It could be concluded that human hepatocellular carcinoma HepG2 cells were more sensitive to both compounds than human breast cancer MD-MB231 cells at the lower IC_50_ value level. 

**Figure 6:**
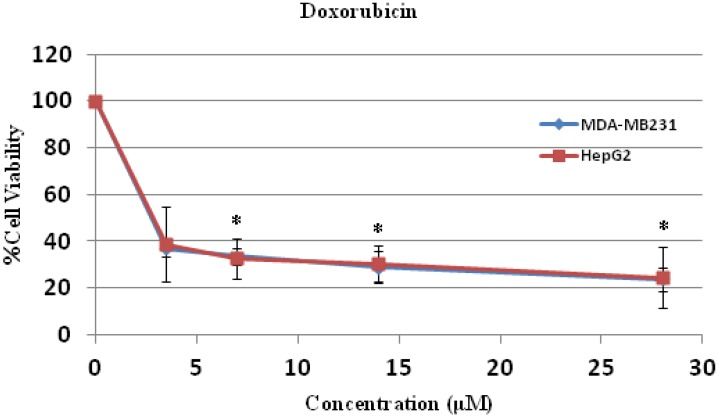
Cell cytotoxicity of doxorubicin on human hepatocellular carcinoma HepG2 and breast cancer MDA-MB231 cells. HepG2 and MDA-MB231 cells were treated with doxorubicin at various concentrations for 24 h and the cell viability were determined by MTT assay. * *p* < 0.05, compared to control.

The anti-cancer activities of both compounds in both cell lines were less potent when compared to doxorubicin (Figure 6, Table 2 and Table 3). The mechanisms of action of doxorubicin are DNA intercalation [12], increased free radical production [13], and inhibition of topoisomerase II progression [14]. Doxorubicin is used for treatment of leukemia, lymphoma and several solid tumors, such as osteosarcoma. It also induces apoptosis and necrosis in healthy tissue of the brain, liver, kidney and heart. The drug influences Bcl-2/Bax apoptosis pathway and caspase activation [15]. However, further study of the mode and mechanism of cell death of compounds **1** and **2** will provide more information of anti-cancer activities of these two compounds and an *in vivo* assay in animal model is required before safe application for cancer treatment in human-beings.

**Table 2:** IC_10_, IC_20_ and IC_50_ levels of compound **1** and **2** in human hepatocellular carcinoma HepG2 cells.

Compound	HepG2					
	IC_10_ (μM)		IC_20_ (μM)		IC_50_ (μM) *	
	MEAN	SD	MEAN	SD	MEAN	SD
**1**	4.21 *^a^	±1.60	8.74 *^a^	±2.22	26.36 *^a^	±5.18
**2**	1.74 *^b^	±0.23	3.46 *^b^	±1.10	12.88 *^b,c^	±2.49
**Doxorubicin**	-	-	-	-	2.21	±1.72

* *p* < 0.05, ^a^ *vs.* control of compound **1**, ^b^ *vs.* control of compound **2**, ^c^ *vs.* IC_50_ level of compound **1**.

**Table 3:** IC_10_, IC_20_ and IC_50_ levels of compound **1** and **2** in human breast cancer MDA-MB231 cells.

Compound	MDA-MB231						
	IC_10_ (μM)			IC_20_ (μM)		IC_50_ (μM) *	
	MEAN		SD	MEAN	SD	MEAN	SD
**1**	5.02 *^a^		±0.53	15.12 *^a^	±1.24	64.75 *^a,c^	±4.45
**2**	3.37 *^b^		±0.88	8.20 *^b^	±2.45	67.06 *^b^	±3.5
**Doxorubicin**		-	-	-	-	1.83	±0.09

* *p* < 0.05, ^a^ *vs.* control of compound **1**, ^b^ *vs.* control of compound **2**, ^c^ *vs.* IC_50_ level of compound **2**.

## 3. Experimental

### 3.1. General Information

Melting points were recorded in degree Celsius (°C) and were measured on a digital Electrothermal melting apparatus. UV spectrum was measured on a Shimadzu 1601 spectrophotometer (Shimadzu, Kyoto, Japan). ^1^H and ^13^C-NMR, ^1^H-^1^H COSY, HMQC and HMBC spectra were recorded with a Unity *plus* 500 spectrometer (Varian Inc, Palo Alto, CA, USA) operating at 500 MHz for ^1^H, and 125 MHz for ^13^C. Low resolution mass spectra were recorded on a Thermo Finnegan Polaris Q mass spectrometer at 70 eV (probe) for EIMS. HRESIMS was obtained by using a Finnigan LC-Q Advantage Thermoquest spectrometer equipped with Xcalibur software (both intruments from Thermo Finnigan, Waltham, MA, USA). IR spectra in KBr disk were recorded on Shimadzu 8900 FTIR spectrophotometer. Column chromatography was conducted on silica gel 60 (Merck 7734, 70–230 mesh). TLC was performed on aluminium backed pre-coated silica gel 60 PF_254_ sheets and detection with using UV detector.

### 3.2. Plant Material

The mixture leaves and twigs of *P. trimera* (Annonaceae) were collected in August 2013 from Chiang Rai Province, the North Thailand. The species was identified by Mr. Narong Nantasean, from The Forest Herbarium, Department of National Park, Wildlife and Plant Conservation, Ministry of Natural Resources and Environment, Bangkok, Thailand. A voucher specimen (BKF.158070) was deposited in the herbarium of this institute.

### 3.3. Extraction and Isolation

The milled dried mixture of leaves and twigs (500 g) was extracted exhaustively in turn with hexane and ethyl acetate-methanol (1:2). The ethyl acetate-methanol extract, on removal of solvent under reduced pressure, gave a black residue (100 g). The extract of the ethyl acetate-methanol extract was fractionated by CC, silica gel, Merck No. 7734, Mesh 70–230 ASTM (600 g, 11 × 20 cm, hexane, hexane-ethyl acetate, ethyl acetate, ethyl acetate-methanol, methanol in order of increasing polarity) to give five fractions (F_1_–F_5_). Fraction F_4_ (31.2 g) was further subjected to CC (silica gel, 200 g, 5 × 16 cm, hexane-ethyl acetate in order of increasing polarity). Eight fractions (A_1_–A_8_) were ultimately obtained on combining the eluates on the basis of TLC. Further, the fraction A_6_ (3.7g) was rechromatographed over silica gel, eluting with dichloromethane, dichloromethane-methanol. The solvents were evaporated to dryness to afford seven subfractions (B_1_–B_7_). The subfraction B_5_ (0.61 g) yielded yellow solid which was recrystallized from methanol/dichlormethane (2:1) to afford orange crystals (26 mg) and identified as 6a,7-dehydro-1,4,5-trimethoxy-7-oxoaporphine (**1**). Further, the mother liquor of B_5_ was recrystallized from dichloromethane (2:1) to give yellow crystals (6.3 mg) of ouregidione (**2**, 6.3 mg). 

*8-Hydroxy-1,4,5-trimethoxy-7-oxoaporphine* (**1**): Orange crystals, m.p. 251–252 °C. UV (MeOH) λ_max_ (log ε): 417(4.58), 317(4.76), 241(5.12), 210(5.08) nm. IR (KBr) v_max _3384, 3249, 2936, 1705, 1658, 1559, 1508, 1458, 1281, 1211 cm^−1^. ^1^H-NMR (DMSO-*d*_6_, 500 MHz) data see Table 1, ^13^C-NMR (DMSO-*d*_6_, 125 MHz) data see Table 1. EI-MS *m/z*: 337 [M]^+^(26), 322(100), 307(6), 294(21), 297(16), 264(17), 91(15). COSY correlations H/H: 2/3, 3/2, 9/10, 10/9, 11, 11/10. HMBC correlations H/C:2/1a, 3; 3/1a, 2; 9/7a, 10, 10a; 10/9, 11, 11a; 11/1a, 7a, 11a; 1OCH_3_/1; 4OCH_3_/4; 5OCH_3_/5; 8OH/ 8. HR-ESI-MS (pos.) *m/z*: 360.0846 ([M+Na]^+^, C_19_H_15_NO_5_Na. calcd. 360.0848.

*1,2,3-Trimethoxy-4,5-dioxo-6a,7-dehydroaporphine* (**2**): Yellow crystals, m.p. 242–243 °C. UV (MeOH) λ_max_ (log ε): 416(4.15), 317(4.34), 214(4.70), 210(4.66) nm. IR (KBr) v_max_ 3420, 2853, 1689, 1658, 1559, 1458, 1211 cm^−1^. ^1^H-NMR (CDCl_3_, 500 MHz) data see Table 1, ^13^C-NMR (CDCl_3_, 125 MHz) data see Table 1. EI-MS *m/z*: 337 [M]^+^(48), 322(100), 306(3), 294(29), 279(23), 264(15), 251(52), 236(21), 180(25), 152(23). COSY correlations H/H: 8/9, 9/10, 10/11. HMBC correlations H/C:7/1b, 8; 8/7, 10; 9/11; 10/9; 11/7a, 9; 1OCH_3_/1; 2OCH_3_/2; 3OCH_3_/3; NH/1b, 4. HR-ESI-MS (pos.) *m/z*: 360.0846 ([M+Na]^+^, C_19_H_15_NO_5_Na. calcd. 360.0849.

### 3.4. Cell Culture

The human hepatocellular carcinoma HepG2 and breast cancer MDA-MB231 cells were cultured in Leibovitz’s L-15 and Dulbecco’s modified eagle media (DMEM), respectively, with 25 mM NaHCO_3_, 20 mM HEPES, 100 units/mL penicillin, 100 μg/mL streptomycin and supplemented with 10% foetal bovine serum. The cell lines were grown at 37 °C in a 5% CO_2_ atmosphere. The cells (5 × 10^3^) were treated with each compound at indicated concentrations for 24 h. The compound **1** and **2** was dissolved in dimethyl sulfoxide (DMSO) as a vehicle and the maximum volume used did not exceed 10 μL/mL of media to avoid the cytotoxicity of DMSO.

### 3.5. MTT Assay

Cell viability was measured by MTT assay (3-(4,5-dimethylthiazol-2-yl)-2,5-diphenyltetrazolium bromide) [16]. In brief, human hepatocellular carcinoma HepG2 and human breast cancer MDA-MB231 cells were seeded in 96-well plates at density of 5,000 cells/well. After 24 h incubation, HepG2 cells and MDA-MB231 cells were treated with or without compound **1** and **2** at various concentrations for 24 h at 37 °C in a humidified 5% CO_2_ atmosphere. Doxorubicin, a chemotherapeutic drug, was used as positive control. Non-treated cells were used as negative control. Then 15 μL (sterile stock solution of 5 mg/mL MTT dye) were added to each well and the solution was incubated for 4 h at 37 °C in a humidified 5% CO_2 _atmosphere. The medium was removed and 100 μL of DMSO were added to each well, and mixed to dissolve the blue crystal formazan. The plate was read at 540 nm, with a reference wavelength of 630 nm, using a microplate reader (Biotek, Winooski, VT, USA) The percentage of cell viability was determined as follows:

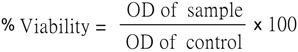



## 4. Conclusions

Compound **1** and **2** displayed anti-cancer activity and have potential for use as anti-cancer drugs in human liver and breast cancer. but the anti-cancer activities of both compounds were less than those of doxorubicin in both cell lines. However, further research in an *in vivo* model is needed before any clinical usage.

## References

[1] Su Y.C.F., Chaowasku T., Saunders M.K. (2010). An extend phylogeny of *Pseuduvaria.* (Annonaceae) with descriptions of three new species and a reassessment of the gerneric status of oreomitra. Syst. Bot..

[2] Su Y.C.F., Saunders R.M.K. (2006). Pseuduvaria. trimera (Craib.). Syst. Bot. Monogr..

[3] Mahmood K., Chan K.C., Park M.H., Han Y.N., Han B.H. (1986). An aporphinoid alkaloid from *Pseuduvaria. macrophylla*. Phytochemistry.

[4] Wirasathien L., Boonarkart C., Pengsuparp T., Suttisri R. (2006). Biological activities of alkaloids from *Pseuduvaria setosa*. Pharm. Biol..

[5] Taha H., Hadi A.H.A., Nordin N., Najmuldeen I.A., Mohamad K., Shirota O., Nugroho A.E., Piow W.C., Kaneda T., Morita H. (2011). Pseuduvarines A and B, two new cytotoxic dioxoaporphine alkaloids from *Pseuduvaria rugosa*. Chem. Pharm. Bull..

[6] Ming Z.S., Shun Z.S., Ning X. (1988). Alkaloids from *Pseuduvaria indochinensis*. Phytochemistry.

[7] Uadkla O., Yodkeeree S., Buayairaksa M., Meepowpan P., Nuntasaen N., Limtrakul P., Pompimon W. (2013). Antiproliferative effect of alkaloids via cell cycle arrest from* Pseuduvaria rugosa*. Pharm. Biol..

[8] Taha H., Arya A., Paydar M., Looi C.Y., Wong W.F., Murthy C.R.V., Noordin M.I., Ali H.M., Mustafa A.M., Hadi A.H.A. (2014). Upregulation of insulin secretion and downregulation of pro-inflammatory cytokines, oxidative stress and hyperglycemia in STZ-nicotinamide-induced type 2 diabetic rats by *Pseuduvaria monticola* bark extract. Food Chem. Toxicol..

[9] Ge Y.W., Zhu S., Shang M.Y., Zang X.Y., Wang X., Bai Y.J., Li L., Komatsu K., Cai S.Q. (2013). Aristololactams and aporphines from the stems of *Fissistigma oldhamii* (Annonaceae). Phytochemistry.

[10] Hsieh T.J., Chang F.R., Chia Y.C., Chen C.Y., Lin H.C., Chiu H.F., Wu Y.C. (2001). The alkaloids of *Artabotrys uncinatus*. J. Nat. Prod..

[11] Wueratne E.M.K., Hatanaka Y., Kikuchi T., Tezuka Y., Gunatilaka A.A.L. (1996). A dioxoaporphine and other alkaloids of two annonaceous plants of sri lanka. Phytochemistry.

[12] Momparler R.L., Karon M., Siegel S.E., Avila F. (1976). Effect of adriamycin on DNA, RNA and protein synthesis in cell-free systems and intact cells. Cancer Res..

[13] Keizer H.G., Pinedo H.M., Schuurhuis G.J., Joenje H. (1990). Doxorubicin (Adriamycin): A critical review of free radical-dependent mechanisms of cytotoxicity. Pharmacol. Ther..

[14] Fornari F.A., Randolph J.K., Yalowich J.C., Ritke M.K., Gewirtz D.A. (1994). Interference by doxorubicin with DNA unwinding in MCF-7 breast tumor cells. Mol. Pharmacol..

[15] Tacar O., Sriamornsak P., Dass C.R. (2013). Doxorubicin: An update on anticancer molecular action, toxicity and novel drug delivery systems. J. Pharm. Pharmacol..

[16] Wudtiwai B., Sripanidkulchai B., Kongtawelert P., Banjerdpongchai R. (2011). Methoxyflavone derivatives modulate the effect of TRAIL-induced apoptosis in human leukemic cell lines. J. Hematol. Oncol..

